# An overview of innovative living arrangements within long-term care and their characteristics: a scoping review

**DOI:** 10.1186/s12877-023-04158-9

**Published:** 2023-07-18

**Authors:** Mara Brouwers, Damien S.E. Broekharst, Bram de Boer, Wim G. Groen, Hilde Verbeek

**Affiliations:** 1 Department of Health Services Research, CAPHRI Care and Public Health Research Institute, Maastricht University, P.O. BOX 616, Maastricht, 6200 MD the Netherlands; 2 Living Lab in Ageing and Long-Term Care, Maastricht, the Netherlands; 3 Department of Public Health and Primary Care, Leiden University Medical Centre, Leiden, the Netherlands; 4 University Network for the care sector South Holland, Leiden University Medical Center, Leiden, The Netherlands; 5 Department of Medicine for Older People, Amsterdam UMC, Vrije Universiteit Amsterdam, Amsterdam, the Netherlands; 6 Amsterdam Public Health Research Institute, Aging & Later Life, Amsterdam, the Netherlands; 7 Amsterdam Movement Sciences, Ageing & Vitality, Rehabilitation & Development, Amsterdam, the Netherlands

**Keywords:** Innovative living arrangements, Innovation, Long-term care, 24-hour care, Nursing home care, Older adults, Scoping review

## Abstract

**Background:**

Within long-term care, a culture change (e.g. focus on increasing autonomy in everyday life) is leading to the development of innovative living arrangements for older adults. Insight into characteristics of innovative living arrangements, which are described as an alternative to regular nursing homes, is lacking. This review aims to provide an overview of innovative living arrangements and to describe their defining characteristics.

**Methods:**

A scoping review was performed following the framework of Arksey and O’Malley. The preferred reporting items for systematic reviews and meta-analyses with extension, for scoping reviews (PRISMA-ScR) was also followed. The databases PubMed, PsycInfo, CINAHL, and Web of Science were searched. Articles, published between 2012 and 2023 were included when they presented an innovative living arrangement as an alternative to regular nursing homes. A thematic analysis was performed, describing the physical, social, and organizational environment of the innovative living arrangements.

**Results:**

Fifty-six articles were identified describing seven types of distinct innovative living arrangements: small-scale living, the green house model, shared housing arrangements, green care farms, dementia villages, group homes, intergenerational living, and an ‘other’ category. The themes included supporting autonomy and creating a small-scale and/or homelike environment, which were emphasized in most innovative living arrangements. Other themes, such as involvement of the community, focus on nature, integration of work tasks, and involvement of family members, were emphasized in a subsection of the described living arrangements. Twenty-eight articles reported on the effects of the innovative living environment on residents, family members, or staff members. Most articles (N = 22) studied resident-related outcomes, focusing mainly on quality of life and aspects of daily life.

**Conclusion:**

More insight into the mechanisms of the social and organizational environments is needed, which may lead to greater transparency and homogeneity regarding the description of living arrangements. This review shows that more knowledge is needed about the potential key elements of innovative living arrangements, especially related to their social and organizational environment. This may provide a better guide for developers within long-term care.

**Supplementary Information:**

The online version contains supplementary material available at 10.1186/s12877-023-04158-9.

## Background

In long-term care, during the past decades, there has been a shift in perspective from a medical approach (e.g. predominant focus on physical care needs) to a more psychosocial approach (e.g. primarily focus on quality of life) [1, 2]. In nursing homes, the traditional focus has primarily been on quality of care and health outcomes. As a consequence there has been an increased orientation towards physical care needs rather than on improving or maintaining quality of life [3]. This medical approach is reflected by the care environment of traditional nursing homes, which are often closed environments, isolated from the community, leading to residents being largely inactive throughout the day [4]. However, residents living in an environment that is less constrained may provide the residents with opportunities to maintain meaningful relationships [5].

The limitations of traditional nursing homes are increasingly being recognized and are leading to a change in culture, one that promotes a resident-directed approach as well as emphasis on quality of life. New insights show that the physical, social, and organizational environment of living arrangements and their interplay are important for achieving positive outcomes for residents [6]. Consequently, alternative living arrangements, which aim to better fit this culture change, have been, and are being, developed. These alternative living arrangements attempt to drastically change the physical, social, and organizational environment to create a better person-environment fit with the aim to improve functioning and quality of life. Examples of alternative living arrangements are, for example, small-scale living (e.g. a joint household with small groups of residents and a fixed team of care staff, centered around household task) [7] or green care farms (e.g. a homelike care environment where agricultural activities are combined with care activities) [8].

The design of the physical environment can be viewed as a therapeutic resource in itself (e.g. facilitating activities indoors and outdoors) that promotes well-being and quality of life among older people [9, 10]. Furthermore, optimizing the social environment and providing person-centred care may also be related to an increase in quality of life [11]. Staff members (e.g. care staff, therapists) play an essential role in supporting the residents’ independence, as they can guide the environment and interactions in a way that stimulates individuality and independence (e.g. empowering residents, avoiding labelling, getting to know residents personally) [12]. Lastly, optimizing the organizational environment (e.g. supportive management, and empowerment of staff members) may relate to a better quality of care in nursing homes [13]. Positive changes in the work environment (e.g. supporting quality of care and ensuring health and personal well-being) seem to result in better teamwork, increased continuity of care, and better resident outcomes [14].

Despite the fast development of alternative living arrangements, research concerning their effectiveness with regard to improving functioning and quality of life is scarce and shows mixed results. There are some indications that innovative living arrangements lead to better outcomes (e.g. greater job satisfaction, social engagement among residents, satisfaction with care of residents, and physical activity of residents) [8, 15–17]. Other articles, however, have not found such effects [17–20].

Authors have described various innovative living arrangements, but their defining characteristics remain unclear. One review has already looked into innovative living arrangements, but the authors focused solely on small-scale living environments [21]. This means that living arrangements offering an alternative to regular nursing homes that are not small-scale were excluded, although they might offer an innovative alternative to regular nursing homes. A complete overview of innovative living arrangements is lacking and more insight is necessary into the components of innovative living arrangements that offer an alternative to regular nursing homes. Therefore, the aim of this scoping review is to provide an overview of the literature concerning innovative living arrangements that are presented as an alternative to regular nursing homes. Furthermore, we aim to describe the defining characteristics and overarching themes addressed by these innovative living arrangements.

## Methods

A scoping review was conducted following the five stages described by Arksey and O’Malley [22]. Furthermore, to increase reliability and transparency, the preferred reporting items for systematic reviews and meta-analyses with extension, for scoping reviews (PRISMA-ScR) was used; see Supporting File 1 for the word file PRISMA-ScR Checklist [23].

### Stage 1: identifying the research question

The following research questions were formulated: What innovative living arrangements are presented in the literature that offer an alternative to regular living arrangements? What are the defining characteristics of these innovative living arrangements?

### Stage 2: identifying relevant studies

To identify potential studies, four electronic databases were searched: PsycInfo, CINAHL, PubMed, and Web of Science. The word file containing the full search string can be found in Supporting File 2. The PCC (population, concept, and context) mnemonic was used to build the search string [24]. The search terms included key terms related to the target group (e.g. older adults), established ‘alternative’ living arrangements (e.g. green care farms), and a combination of facility names (e.g. nursing home) and terms related to innovation. Additionally, for each of these key terms, the plural tense and conjugates were also included. A librarian checked and finalized the search string for all included databases. Our first search was performed on 5 July 2022. However, to make sure the review was up-to-date, an update was performed on 22 May 2023. Additionally, reference lists of all included articles and reference lists of reviews were searched to identify additional potentially relevant articles. When an article referred to another article for the definition of a specific living arrangement and it was traceable and relevant, the article could still qualify.

### Stage 3: study selection

Articles published in the Dutch or English language between 2012 and May 2023 were included. As the purpose of this review was to identify recent developments within the long-term care landscape, this review focuses on articles published in the last decade. Articles were included if they: (1) consisted of original research articles describing primary data; (2) described a living arrangement as an alternative to regular nursing homes that offer 24-hour care; (3) presented a description of an innovative living arrangement; (4) described an innovative living arrangement that offers 24-hour care (psychogeriatric as well as somatic care needs) to older adults with complex care needs. An article was excluded when: (1) it did not present original data (e.g. opinion paper); (2) the innovative living arrangement described did not offer 24-hour long-term care; (3) the described innovative living arrangement was not yet operational; (4) the living arrangement focused on short-term stay, rehabilitation, or hospital stay. See Table 1 for overview of the inclusion and exclusion criteria.

**Table 1:** Inclusion and exclusion criteria

PCC element	Inclusion criteria	Exclusion criteria
**Population**	Older adults with a complex care need, in need of 24-hour care.	Participants that do not have a complex need, are not in need of 24-hour care, or are young.
**Concept**	Long-term care facilities that offer 24-hour care to older persons with a complex care need. This entails 24-hour care in both psychogeriatric, as well as somatic care needs. Long-term care facilities that offer long-term care, so care for an extensive period of time, to older persons. Long-term care facilities that are presented as different/innovative in comparison with traditional 24-hour long-term care. The facility has to be operational.	Long-term care facilities that do not offer long-term 24-hour care (in psychogeriatric needs and ADL assistance) or care concepts that offer 24-hour care to adults or youth. Long-term care facilities that provide acute (or secondary acute) care, such as hospitals and revalidation centers. Traditional long-term care facilities or implemented interventions within existing traditional long-term care facilities. Long-term care facilities that are not yet operational and present possible frameworks or best practices.
**Context**	Long-term care facilities for older adults	Short-term care, such a rehabilitation or hospital stay, or long-term care for a group, other than older adults in need of 24-hour care.

All articles were imported into EndNote [25] and Rayyan review managing software [26], which were used for the remainder of the screening process. Both the first author (MB) and a fellow researcher (DB) independently screened the articles based on their titles and abstracts. Before the actual screening process, about 50 articles were test-screened to make sure all in- and exclusion criteria were clear. In the second phase, both researchers independently screened the full-text articles and again determined whether the articles met the eligibility criteria. Any discrepancies between their outcomes were discussed and resolved by re-evaluating them together against the criteria and, if necessary, by discussing the articles with the entire research team to reach a consensus.

### Stage 4: charting the data

A data extraction form was developed. The form included: (1) an extensive description of the innovative living arrangement: name; location; the number and characteristics of residents; the number of units/buildings; a general description; and a description of the physical, social, and organizational environment; (2) the main characteristics of the article: title; date; authors; research question(s); and study design (i.e. observational, quasi-experimental, experimental, and qualitative); (3) sample and sample characteristics; data collection method; and description of data analyses; (4) the primary study outcomes.

### Stage 5: collating, summarizing, and reporting the results

Data analysis consisted of conducting a thematic analysis [27] of the descriptions of the alternative living arrangements. The data was analyzed following the six phases of Nowell, Norris [27]. First, the researchers familiarized themselves with the included articles by reading them thoroughly. Second, an extensive description of the innovative living arrangements was created based on the various descriptions in the individual articles. Specifically, the physical, social, and organizational environments were described and presented in a table. The concepts were grouped into overarching living arrangements, based on overlap in their description. Then the components mentioned in the descriptions were coded. Third, these codes were grouped into overarching themes (e.g. homelike environment, community involvement, etc.). Fourth, all themes were discussed with the last author. Fifth, based on these discussions, the themes were further defined and renamed in such a way to capture the essence of the theme. Last, all themes were described in a report, leading to the results section.

## Results

**Fig. 1:**
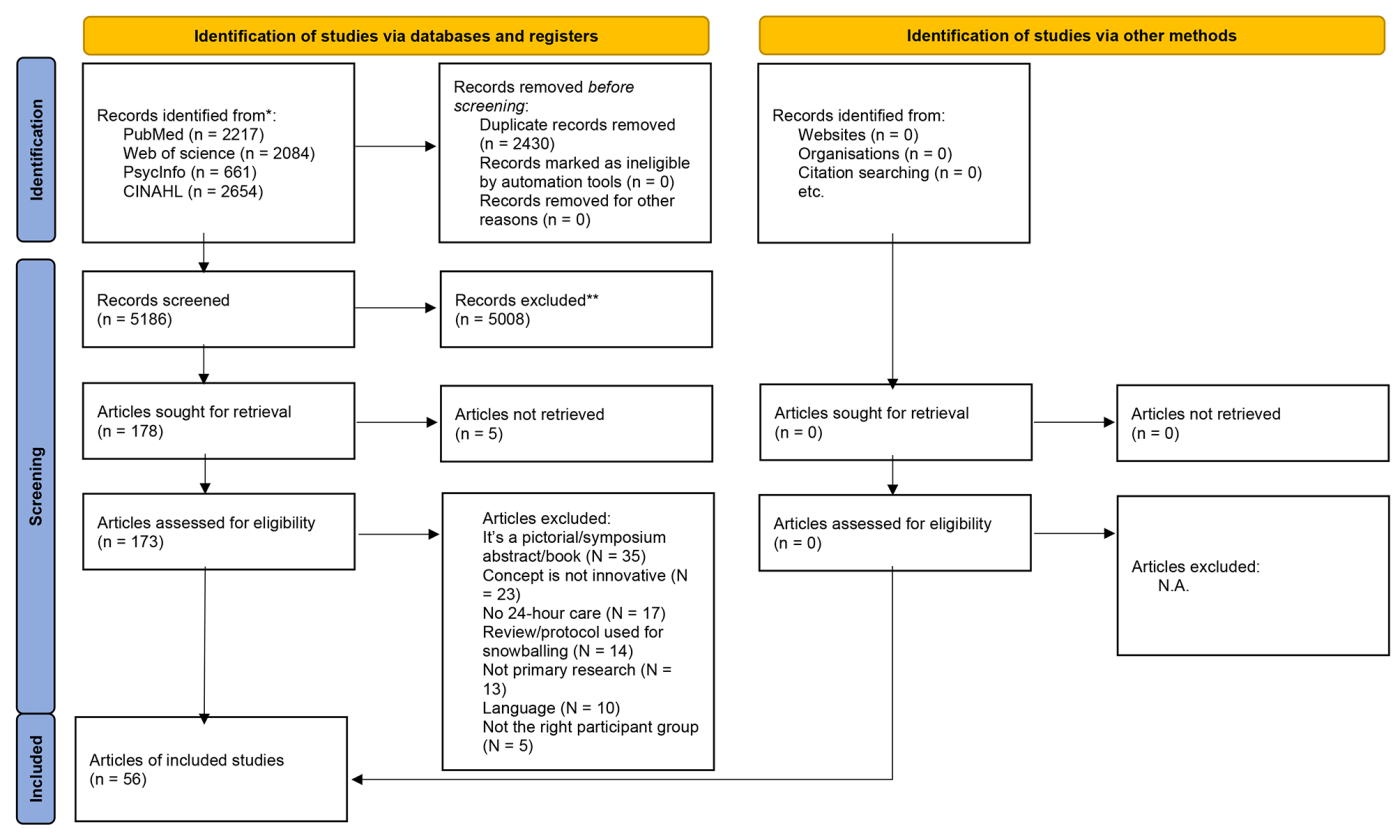
Flowchart data-selection process

In total, 7616 articles were identified. After removal of all duplicates, 5186 articles were included for screening. Figure 1 presents the PRISMA 2020 flow diagram [28] showing the search results. Title/Abstract screening has led to 173 articles eligible for full-text screening. Evaluation of these articles according to the inclusion and exclusion criteria resulted in the inclusion of 56 articles suitable for the scoping review. The snowballing method did not lead to identification of new articles.

Finally, 56 articles were selected to answer our research question. These articles described a total of seven distinctive innovative living arrangements: (1) small-scale living [15, 16, 18, 29–39], (2) the green house model [40–47], (3) shared housing arrangements [48–54], (4) green care farms [20, 55–61], (5) dementia villages [40, 62–66], (6) group homes [67–69], and (7) intergenerational living [70–72]. Some articles, however, could not be grouped into one overarching living arrangement because they did not describe the same overarching elements (a household model of residential aged care [73], household model units [74, 75], intensive service housing [76], a non-traditional residential care facility [77], the Woodside place model [78], a small-scale homelike unit [79], and a homelike dementia care facility [80]) and were described as an ‘other’ category. An overview of the identified types of innovative living arrangements and their characteristics is provided in Table 2.

**Table 2:** An overview of the identified types of innovative living arrangements and their characteristics

Living arrangement	Articles; Country	Number of residents	Articles design	General description	Physical environment	Social environment	Organizational environment
**Small-scale living**	14 Articles [15, 16, 18, 29–39]; the Netherlands, Belgium	6–16 residents per household	(Quasi-)experimental quantitative (N = 11) [15, 16, 31–39], observational quantitative (N = 1) [29], Qualitative (N = 2) [18, 30]	A small group of older people forming a household together in a home-like environment. Normalization of daily life is emphasized, by supporting their capabilities and focusing on meaningful activities (e.g. social participation and household chores).	The facility resembles an archetypal house, with homelike features (e.g. a kitchen, living room, single bedrooms, and an entrance).	Daily household tasks are centred around activities of daily life (e.g. doing laundry, preparing meals together, and cleaning). Daily life is largely determined by the residents, family caregivers, and nursing staff. There is more personal contact due to the fixed team of nursing staff and person-centred care.	The tasks of staff members are integrated, meaning they carry out domestic, social, and recreational tasks in addition to care tasks. There is often a fixed team of staff members that take care of the residents.
**Green house model**	8 Articles [40–47]; United States	8–12 residents per household	(Quasi-)experimental quantitative (N = 3) [44, 45, 47], Qualitative (N = 5) [40–43, 46]	The green house model was developed by William Thomas and is based on the Eden Alternative in the US. The model emphasizes a homelike environment and significantly transformed care staff roles.	This model includes small-scale and homelike facilities, with a family-style physical environment.	Normalized daily activities are promoted, such as doing laundry or cooking, and green house homes encourage and support autonomy.	Certified nursing assistants, referred to as the ‘Shahbazim’, are at the centre of the green house model. They work in self-managed teams and have integrated tasks (e.g. resident care, household chores, and staff scheduling). Nurses have a more visiting and clinical role. The so-called ‘Guide’, whose office is outside the home, often guides staff members. Green houses are often opened alongside a large nursing home, called a ‘legacy home’.
**Shared housing arrangements**	7 Articles [48–54]; Germany	6–8 residents	(Quasi-)experimental quantitative (N = 5) [49–51, 53, 54], Observational quantitative (N = 1) [52], Qualitative (N = 1) [48]	This approach is an alternative to regular nursing homes, where a small group of older people in need of care live together in a small-scale, homelike living facility.	It is a regular apartment building, usually in an urban setting, with a small-scale, homelike appearance.	The daily routines focus on ‘family living’ and living as self-determinedly and normally as possible, by doing household chores together. Family is also involved by participating in the daily life in shared housing arrangements and acting as legal representatives. As shared housing arrangements are located in residential districts, residents are encouraged to participate in the social life of the community.	Shared housing arrangements are not connected with residential care, as care is provided by community care services. Shared housing arrangements are long-term, meaning that residents do not have to move to a nursing home when their care needs are increasing.
**Green care farms**	8 Articles [20, 55–61]; The Netherlands	6–60 residents	(Quasi-)experimental quantitative (N = 3) [20, 56, 58], Qualitative (N = 5) [55, 57, 59–61]	In green care farms, agricultural activities are combined with care services for several groups, such as older people with dementia. They provide care in a small-scale, homelike environment on the terrain of a farm.	Green care farms offer a familiar and small-scale, homelike environment. The facility often resembles an archetypal house. Animals, plants, and natural aspects are present.	Green care farms focus on meaningful activities in everyday life as well as autonomy. Residents are encouraged to participate in a range of activities that are meaningful and stimulating, such as domestic (e.g. cooking, doing laundry), work-related (e.g. feeding animals, gardening), social (e.g. coffee break), and recreational (e.g. reading, playing games) activities. Due to free access to outdoor areas, residents always have the opportunity to go outside.	Green care farms differ in the degree of farming and care, meaning that some farms are actual farms with a profitable production. For other locations, care is the main source of income. The farmers are often personally involved in developing the care vision and motivating staff members. Staff members have integrated tasks
**Dementia village**	6 Articles [40, 62–66]; The Netherlands, Denmark, Germany	52–152 Residents, but divided into smaller group homes	Qualitative (N = 5) [40, 62–65] (Quasi-)experimental quantitative (N = 1) [66]	A dementia village introduces a non-institutional village-type of accommodation, often located in a mid-sized town. It originates from the dementia village of Hogeweyk in the Netherlands. It aims to create an environment that enables residents to live as normally as possible, while still feeling part of the local community.	The village is developed in such a way as to resemble a familiar environment for the residents (e.g. a high street, town square, supermarket, activity centre, connecting paths between residences, and gardens).	Residents are matched and placed into the same home, based on their background. Autonomy is encouraged by normalizing daily life and being able to choose what to do during the day.	The environment is designed to protect, but not restrict the residents. Professional and institutional features are hidden as well as possible. Staff members and caregivers wear clothing that fits the lifestyle, so no uniforms are worn.
**Group homes**	3 Articles [67–69]; Japan	5–12 residents	(Quasi-)experimental quantitative (N = 2) [67, 69], Observational quantitative (N = 1) [68]	A group-living based facility for residents with dementia.	A small-scale and homelike environment in a familiar community.	There is particular attention paid to familiar relationships and each patient’s lifestyle. Staff members live together with residents.	At least one staff member per three residents is allocated as full-time personnel.
**Intergenerational living**	3 Articles [70–72]; the Netherlands	166 residents	Qualitative (N = 3) [70–72]	In this living environment, students and older residents live together. This living environment is based on social reciprocity and a feeling of community.	The setting is a large institutional building.	There is one student for every 25 residents in the intergenerational living environment. The students act as ‘good neighbours’ and share experiences and perform activities with the older residents. In return, older residents share their experiences. Reciprocity is key within this vision. The location has a central place in the community. Older people, students, and others in need of support live together and the broader community is welcome as well.	Students who study nursing or medicine are not allowed, to promote a more natural environment. In exchange for free accommodation, students perform 30 hours per month of social-type work. Caregivers and volunteers are in charge of care services and the annual budget. There is a ‘yes’-culture within the organization, to motivate all those involved to propose ideas and solutions.
**Other**:							
** Household model of residential aged care** [73]	Australia	16–30 residents per household	Qualitative	A homelike environment that focuses on positive ageing and maintaining a sense of ‘self’.	A home-like appearance with a kitchen, dining room, and self-contained apartments.	There are no fixed schedules. Continuous access to food and the ability to choose is central to this model.	The staff member responsible for coordinating the household is named ‘Homemaker’. Staff members are encouraged to work autonomously and perform integrated tasks.
** Household model units** [74, 75]	2 Articles; Ireland	16–18 residents	(Quasi-)experimental quantitative (N = 2)	This homelike environment emphasizes autonomy and privacy.	This homelike environment features open plan areas.	There are no fixed schedules and autonomy is encouraged by offering choices.	There is a new staff role, called ‘Homemaker’. This person’s role is defined by the kitchen and household tasks. He/she has a constant monitoring presence in the open plan area.
** Intensive service housing** [76]	Finland	/	Qualitative	Homelike housing units located in intensive service housing facilities. ‘Home’ is a strongly emphasized word.	The unit is decorated with the residents’ belongings. Movement is restricted.	The central idea of this arrangement is homemaking, thus prioritizing homelike and domestic-style care.	The resident pays for all services (e.g. Rent, care, meals, cleaning) separately.
** Non-traditional residential care facility** [77]	Australia	/	Qualitative	This approach utilizes principles of environmental design to create a dementia-friendly environment.	The building has an open floor plan and a homelike, domestic appearance.	Employees initiate spontaneous group activities, instead of planning them.	/
** Woodside place model** [78]	Canada	12 residents per Household	(Quasi-)experimental quantitative	This approach provides a supportive and secure homelike environment, with a focus on supporting autonomy.	The environment is a small and homelike setting, with a household with a small dining room, kitchen, and bedrooms as the core component.	The social environment emphasizes a normal way of living, adapted to cultural values	/
** small-scale homelike unit** [79]	Canada	12 residents	(Quasi-)experimental quantitative	This approach creates a small-scale homelike unit.	The environment includes a short corridor with single bedrooms and a single-loaded floor plan.	/	In the daytime, 1.5 nurses and two care aides work at the location.
** Homelike dementia care facility** [80]	China	20 residents	Qualitative	This approach aims to create a homelike environment for people with dementia	This environment includes a residential area, activity areas, and common areas	Residents can engage in various activities, including homelike activities (e.g. household chores, exercising).	/

### Study characteristics

Studies on innovative living arrangements were performed in the following countries: the Netherlands (N = 26), the United States (N = 8), Germany (N = 8), Belgium (N = 4), Japan (N = 3), Denmark (N = 2), Australia (N = 2), Ireland (N = 2), Canada (N = 2), Finland (N = 1), France (N = 1), and China (N = 1). The included articles consisted of 25 qualitative studies, 3 observational quantitative studies, and 28 (quasi-)experimental quantitative studies. In general, 28 quantitative studies were performed, focusing on the effects in small-scale living (N = 11), the green house model (N = 3), shared housing arrangements (N = 5), green care farms (N = 3), group homes (N = 2), and the ‘other’ category (N = 5). Most articles focused on quality of life (N = 10), physical health (N = 8), job characteristics (N = 6), and variables related to social engagement/activities of daily life (N = 6).

### Themes

By analysing the data based on the physical, social, and organizational environment of the described innovative living arrangements, the following themes emerged: promoting autonomy, small-scale/homelike environment, involvement of community, focus on nature, integration of work tasks, and involvement of family members. These themes describe the similarities and differences among the innovative living arrangements and lead to a clear overview of the characteristics of the described living arrangements.

#### Promoting autonomy

Six out of seven arrangements and two articles in the ‘other’ category (household model of residential aged care and household model units) emphasized the importance of promoting autonomy. In most arrangements, autonomy is fostered by normalizing daily life and offering choice, meaning that the residents live their lives as normally as possible, minimizing rigid routines, that are often seen in more regular nursing homes. This is often encouraged by involving residents in daily household tasks. Centering the daily lives of residents around daily routines is applied in small-scale living, the green house model, and shared housing arrangements. Green care farms also focus on daily routines but emphasize nature and farm-based daily activities (e.g. feeding the animals and gardening). Although almost all living arrangements emphasize the importance of promoting autonomy, a clear definition of what autonomy entails is often missing. Furthermore, more information on how they exactly aim to promote autonomy is often not provided.

#### Small-scale homelike environment

Five out of seven living arrangements focus on creating a small-scale and/or homelike environment. Small-scale living, the green house model, shared housing arrangements, green care farms, group homes, and two living arrangements of the ‘other’ category (the Woodside place model, and a small-scale homelike unit) focus on creating both a small-scale and homelike environment. Dementia villages and the remaining four living arrangements of the ‘other’ category focus solely on creating a homelike environment, although dementia villages implicitly suggest that there is also small scale-ness, as residents are divided into smaller group homes in the village. The interpretation of small scale-ness seems to differ among, but also within, living arrangements. They differ in group size from 6 to 16 residents, showing diversity in the considered appropriate number of residents for small scale-ness. Most living arrangements describe at-homeness in a similar manner, as the facility often resembles an archetypal house with a kitchen, living room, and self-contained apartment. The articles mostly focus on the physical appearance of at-homeness, but the authors provide little to no information about the role of the social and organizational environment in creating a homelike atmosphere.

#### Integration of work tasks

Three living arrangements (small-scale living, green house model, and green care farm) and two articles of the ‘other’ category (household model of residential aged care and household model units) focus on integrated tasks of staff members. This entails that staff members not only perform care-related tasks, but also domestic, social, and recreational tasks. The living arrangement that stands out here is the green house model, as this arrangement has created new care roles, named the Shahbazim and the Guide. The Shahbazim are the direct care staff who are responsible for a broad array of tasks (e.g. resident care, household tasks, activities, and staff scheduling). The Guide, with an office outside the green house, acts as a coach and supervises the Shahbazim in all non-clinical aspects. The living arrangements that do not specifically mention the integration of tasks of staff members do often mention a de-institutionalized way of working (e.g. not wearing uniforms, hiding institutional aspects such as a nursing station, having a fixed team of staff members).

#### Involvement of the community

Two living arrangements emphasize involvement of the community. Within intergenerational living older residents live together with students, and the community and others in need of support are also encouraged to visit. Shared housing arrangements stress the importance of community volunteers and their social involvement in caring for the residents. Other living arrangements do not explicitly describe the involvement of the neighbourhood. These arrangements focus more on creating community within the living arrangement, by creating a family-like household of residents and staff members. This is broadened in dementia villages, where an entire ‘village-type’ accommodation is created to create a community on its own for the residents. The focus is more on the inside community, rather than the community outside the dementia village.

#### Focus on nature

Two living arrangements (i.e. green care farms and dementia villages) explicitly mention the focus on nature. In green care farms, nature has a prominent role. The daily lives of residents revolve around agricultural activities. There are animals and plants present, and the facility is often part of some sort of farm. Going outside and being involved with nature is encouraged in green care farms. Articles describing a dementia village also mention the presence of a park but residents are not explicitly encouraged to be engaged with nature.

Within different small-scale living arrangements (small-scale living, the green house model, and group homes), there are individual differences in the use of nature. Some articles describe the presence of an outdoor space and/or garden, while other articles do not mention this. In these living arrangements, the presence of nature seems to be location-specific, rather than concept-specific. Shared housing arrangements, on the other hand, seem to focus more on urban locations, and do not focus on, or mention, nature.

#### Involvement of family members

A few articles explicitly describe the role of family in the living arrangement. In shared housing arrangements, there is a strong emphasis on the involvement of family members. They serve as key people for the residents and nursing staff. For example, some articles describe that they are involved in all aspects of care provision, decision-making, household tasks, and activities [49–51]. In small-scale living, some articles describe that family members determine the organization of daily life together with the residents and staff members [15, 16, 18, 31, 39]. None of the other living arrangements explicitly describe the family involvement.

### Effectiveness

Twenty-eight articles reported on the effects of the alternative living environment on either residents, family members, or staff members. Most articles (N = 22) studied resident-related outcomes, with eight articles focusing on quality of life. The articles showed mixed results, from no effect on quality of life (N = 5) [18, 20, 50, 53, 54], to effects only on sub-scales of the quality of life questionnaires used (N = 2) [34, 37]. Only one article showed a significant effect on quality of life of residents when family members were actively involved within shared housing arrangements [49]. Other articles focused mainly on outcomes related to the daily/social life of residents. These articles include a diverse number of outcome measures. Most articles showed a positive effect on some – but not all – outcome measures [16, 29, 35, 58, 74, 75, 79]. Four articles studied staff member–related outcomes, all focusing on job characteristics. Three articles showed positive outcomes for job-related characteristics (e.g. stress, burnout, and job satisfaction) [15, 31, 32], and one showed a negative effect on fatigue and no effect on the other outcomes [33]. Two articles studied family-related outcomes, showing that family members felt less burdened and more satisfied with the alternative living environment [18, 35].

## Discussion

This scoping review has presented an overview of alternative living arrangements within long-term care and their core characteristics. Seven overarching living arrangements have been identified: small-scale living, the green house model, shared housing arrangements, green care farms, dementia villages, group homes, intergenerational living, and an ‘other’ category. Emerging themes of these living arrangements are the importance of stimulating and supporting autonomy. Furthermore, most living arrangements focus on creating a small-scale and/or homelike environment. The other themes – involvement of the community, focus on nature, integration of tasks staff members, and involvement of family members – are emphasized in some of the described living arrangements. Quality of life, and outcomes related to daily/social life have been the most studied measures.

In most articles, the main focus is on the physical environment, where the features of the indoor and outdoor areas are often described in detail. For example, the authors describe what makes a location ‘homelike’, including the physical features (e.g. the furniture is recognizable and placed in a manner that facilitates social interaction, there are animals present, personal belongings are present). Even though recent insights highlight the importance of the social and organizational environment [6], most descriptions of alternative living arrangements lack specific information on these components. It is important to have congruence between the physical, social and organizational environment to promote optimal well-being and daily functioning [6]. Only describing and focusing on the physical environment gives an incomplete overview of the functioning of living arrangements. When looking at the example of at-homeness, research shows that it is more than just the physical environment. It also entails a feeling of autonomy, feeling safe and respected by staff members and other residents, and building meaningful relationships [81]. A meaning of home is a combination of physical, social, and individual aspects [82], showing that next to a home-like physical environment, older adults perceive ‘feeling at home’ as being able to preserve their personal identity, experiencing continuity in life, feelings of belonging, and being active [83]. This illustrates that the social and organizational features are just as important as physical features in understanding how a living arrangement operates.

One of the core themes throughout most living arrangements is the promotion/support of autonomy. Most articles describe that autonomy is supported by letting residents determine how to spend their daily lives and performing meaningful activities, such as doing household chores. The articles do not define autonomy explicitly and how support of autonomy is operationalized within alternative living arrangements. Autonomy focuses on independence and self-determination, meaning that independence of action, speech, and thought is important [84]. For residents with complex care needs, autonomy is not always a given, due to their increase in care dependency. Nevertheless, research focusing on autonomy within long-term care emphasizes the importance of relational autonomy, meaning that the social environment plays an important role in facilitating autonomy [85]. How the social environment facilitates autonomy within the included living arrangements, however, remains rather abstract and unclear. Despite the fact that living arrangements stress the importance of autonomy, researchers have not explained how to apply this philosophy to residents with complex care needs in practice. What is needed, for example, is a description of what role the staff members have in supporting autonomy and how they can operationalize this in practice. Relationships between staff and residents can either facilitate or inhibit autonomy [85], and more and more practices to facilitate autonomy, such as reablement (which focuses on mitigating the impact of dementia on functioning and dementia) are being developed [86].

Innovation is often associated with the use of technology. Notably, the role of technology is not described in the included articles concerning innovative living arrangements. The presence of technology in long-term care is becoming more prominent; examples include socially assistive robots, and technology to prevent falls and to ensure safety [87, 88]. Governmental, academic, and private organizations are increasingly developing and deploying technology in ageing services [89]. These technologies range from sensors (e.g. bio-sensors and motion sensors) to virtual reality and remote communication possibilities [89]. More insight is needed regarding the role of technology in these living arrangements –for example, enabling the autonomy of residents to move around by using GPS – and why this aspect is not represented properly in the published literature. A possible explanation is that technology in long-term care is still in a rather exploratory phase. The available research concerning technology shows mixed results in terms of effectiveness [87, 88]. Another possible explanation is that we did not explicitly include terms related to technology in our search string, meaning that we might not have found these articles.

A few limitations of this scoping review should be addressed. First, there is a possibility that the search string did not identify all relevant living arrangements, as terminology varies among articles and living arrangements. Second, articles that are not written in English or Dutch/Flemish were excluded, and grey literature was excluded as well, meaning that some relevant living arrangements might not have been included. Third, we only performed backward snowballing, meaning that we did thus not perform forward snowballing and might have missed relevant articles. Fourth, there is a delay between current practice and the literature, meaning there may be other innovative arrangements available or tested that we could not capture with our literature-based review. Lastly, there is a lot of variety in the amount of information provided among the identified arrangements. Some articles present a clear case study with an extensive description of the living arrangements, while others provide minimal description. This reality made it challenging to extract data and to identify the core themes among the living arrangements.

Although there is an increasing interest in innovative living arrangements, much is still unknown. This review has attempted to provide an overview of innovative living arrangements described in the literature and to describe their core characteristics. The results of this scoping review show that living arrangements using the same terminology can still differ quite a lot in operationalization. Greater clarity should be provided about the underlying physical, social, and organizational mechanisms that define an alternative living arrangement. There is a lot of diversity within alternatives for regular nursing homes. Future effectiveness studies are easier to carry out when descriptions of key elements are transparent. Furthermore, gaining insight into the physical, social, and organizational environment of alternative living arrangements will improve the knowledge of developers in long-term care, providing them with support when developing alternative living arrangements.

## Conclusion

For future research, it is important to identify the working components of not only the physical environment, but also the social and organizational components to broaden our understanding of the underlying working mechanisms of alternative living arrangements. When developing, it is key to not only think about the physical environment, but also consider how to operationalize the vision of the living arrangement. Developers should consider what role staff members will have, how the social surrounding will be utilized, and how care should be organized within the physical setting. When evaluating an innovative living arrangement, it is key to not only consider the physical environment, but to consider the social and organizational environment as well. Therefore, a better understanding of the mechanisms of alternative living arrangements may provide a better guide for developers within long-term care. This review shows that more knowledge is needed about potential key elements of innovative living arrangements, especially related to their social and organizational environment.

## Electronic supplementary material

Below is the link to the electronic supplementary material.

Supplementary Material 1

Supplementary Material 2

## Data Availability

All data generated or analysed during this study are included in this published article and its supplementary information files.

## List of Abbreviations

PRISMA-ScR: preferred reporting items for systematic reviews and meta-analyses with extension, for scoping reviews.
