# Supplementary material for: An overview of innovative living arrangements within long-term care and their characteristics: a scoping review
Source: BMC Geriatr. 2023 Jul 18;23:442. doi: 10.1186/s12877-023-04158-9 (PMC10355008; doi:10.1186/s12877-023-04158-9)
Supplement: Supplementary file 2 — Supplementary Material 2 [file 12877_2023_4158_MOESM2_ESM.docx]

**Search string PubMed**

**Section 1:**

("Aged"[MeSH] OR "Dementia"[MeSH] OR "Health services for the aged"[MeSH] OR "Geriatrics"[MeSH] OR "Geriatric Psychiatry"[MeSH] OR "Geriatric Nursing"[MeSH] OR "Old adult*"[tiab] OR "Old people"[tiab] OR "Older adult*"[tiab] OR "Older people"[tiab] OR "elder*"[tiab] OR "Dementia"[tiab] OR "Senior*"[tiab] OR "Geriatric*"[tiab] OR "ages 65"[tiab] OR "aged 65"[tiab] OR "65 and over"[tiab] OR "ages 70"[tiab] OR "aged 70"[tiab] OR "70 and over"[tiab] OR "ages 75"[tiab] OR "aged 75"[tiab] OR "75 and over"[tiab] OR "ages 80"[tiab] OR "aged 80"[tiab] OR "80 and over"[tiab] OR "ages 85"[tiab] OR "aged 85"[tiab] OR "85 and over"[tiab] OR "ages 90"[tiab] OR "aged 90"[tiab] OR "90 and over"[tiab] OR "eldest"[tiab] OR "frail*"[tiab] OR "geriatri*"[tiab] OR "old age*"[tiab] OR "oldest old*"[tiab] OR "senium"[tiab] OR "very old*"[tiab] OR "septuagenarian*"[tiab] OR "octagenarian*"[tiab] OR "nonagenarian*"[tiab] OR "centarian*"[tiab] OR "centenarian*"[tiab] OR "supercentenarian*"[tiab] OR "older subject*"[tiab] OR "older patient*"[tiab] OR "older age*"[tiab] OR "older men"[tiab] OR "older male*"[tiab] OR "older woman"[tiab] OR "older women"[tiab] OR "older female*"[tiab] OR "older population*"[tiab] OR "older person*"[tiab])

AND

**Section 2A:**

("Group homes"[MeSH] OR "Group living"[tiab] OR "Group dwelling*"[tiab] OR "Collective living"[tiab] OR "Homelike"[tiab] OR "Home like"[tiab] OR "Small scale"[tiab] OR "Smallscale"[tiab] OR "Small unit*"[tiab] OR "Care farm*"[tiab] OR "Green care"[tiab] OR "Green dementia care"[tiab] OR "Social farm*"[tiab] OR "Green hous*" [tiab] OR "dementia friendly"[tiab] OR "homeshar*"[tiab] OR "Dementia villag*"[tiab] OR "Cohous*"[tiab] OR "shared hous*"[tiab] OR "communal liv*"[tiab])

OR

**Section 2B:**

("Housing for the Elderly"[Mesh] OR "Institutionalization"[MeSH] OR "Long-Term Care"[MeSH] OR "Geriatric Nursing"[MeSH] OR "Residential Facilities"[MeSH] OR "Environment Design"[MeSH] OR "Nursing homes"[MeSH] OR "24-hour care facilit*"[tiab] OR "care home*"[tiab] OR "care institution*"[tiab] OR "extended care facilit*"[tiab] OR "geriatric center*"[tiab] OR "geriatric centre*"[tiab] OR "geriatric facilit*"[tiab] OR "geriatric home*"[tiab] OR "geriatric institution*"[tiab] OR "geriatric unit*"[tiab] OR "group home*"[tiab] OR "home for the aged"[tiab] OR "institutionalized elderly"[tiab] OR "intermediate care facilit*"[tiab] OR "long term care"[tiab] OR "LTCF*"[tiab] OR "nursing center*"[tiab] OR "nursing centre*"[tiab] OR "nursing facilit*"[tiab] OR "nursing home*"[tiab] OR "nursing institution*"[tiab] OR "nursing unit*"[tiab] OR "old age home*"[tiab] OR "residential care"[tiab] OR "residential facilit*"[tiab] OR "skilled nursing facilit*"[tiab] OR "Homes for the elderly"[tiab] OR "Home for the elderly"[tiab] OR "Home for elderly"[tiab] OR "Homes for elderly"[tiab] OR "assisted living facilit*"[tiab] OR "continuing care retirement communit*"[tiab] OR "old folks home*"[tiab] OR "old folks' home*"[tiab] OR "old people's home*"[tiab] OR "rest home*"[tiab] OR "retirement facilit*"[tiab] OR "retirement home*"[tiab] OR "age friendly communit*"[tiab] OR "Livable communit*"[tiab] OR "Intergenerational living"[tiab] OR "Intergenerational housing"[tiab] OR "Senior hous*"[tiab] OR "special care unit*"[tiab] OR "Special care facilit*"[tiab])

AND

("Organizational innovation"[MeSH] OR "Diffusion of Innovation"[MeSH] OR "Inventions"[MeSH] OR "Knowledge Management"[MeSH] OR "Health Care Reform"[MeSH] OR "innovat*"[ti] OR "Original"[ti] OR "visionar*"[ti] OR "chang*"[ti] or "alternative*"[ti] OR "modernization*"[ti] OR "modernisation*"[ti] OR "cutting edge"[ti] OR "leading edge"[ti] OR "inventi*"[ti] OR "Ingenious"[ti] OR "reform*"[ti])

**Search string EBSCOHOST: CINAHL**

**Section 1:**

(MH "Aged+" OR MH "Dementia+" OR MH "Health Services for the Aged" OR MH "Geriatrics" OR MH "Geriatric Psychiatry" OR MH "Gerontologic Nursing+" OR TI "Old adult*" OR TI "Old people" OR TI "Older adult*" OR TI "Older people" OR TI "elder*" OR TI "Dementia" OR TI "Senior*" OR TI "Geriatric*" OR TI "ages 65" OR TI "aged 65" OR TI "65 and over" OR TI "ages 70" OR TI "aged 70" OR TI "70 and over" OR TI "ages 75" OR TI "aged 75" OR TI "75 and over" OR TI "ages 80" OR TI "aged 80" OR TI "80 and over" OR TI "ages 85" OR TI "aged 85" OR TI "85 and over" OR TI "ages 90" OR TI "aged 90" OR TI "90 and over" OR TI "eldest" OR TI "frail*" OR TI "geriatri*" OR TI "old age*" OR TI "oldest old*" OR TI "senium" OR TI "very old*" OR TI "septuagenarian*" OR TI "octagenarian*" OR TI "nonagenarian*" OR TI "centarian*" OR TI "centenarian*" OR TI "supercentenarian*" OR TI "older subject*" OR TI "older patient*" OR TI "older age*" OR TI "older men" OR TI "older male*" OR TI "older woman" OR TI "older women" OR TI "older female*" OR TI "older population*" OR TI "older person*" OR AB "Old adult*" OR AB "Old people" OR AB "Older adult*" OR AB "Older people" OR AB "elder*" OR AB "Dementia" OR AB "Senior*" OR AB "Geriatric*" OR AB "ages 65" OR AB "aged 65" OR AB "65 and over" OR AB "ages 70" OR AB "aged 70" OR AB "70 and over" OR AB "ages 75" OR AB "aged 75" OR AB "75 and over" OR AB "ages 80" OR AB "aged 80" OR AB "80 and over" OR AB "ages 85" OR AB "aged 85" OR AB "85 and over" OR AB "ages 90" OR AB "aged 90" OR AB "90 and over" OR AB "eldest" OR AB "frail*" OR AB "geriatri*" OR AB "old age*" OR AB "oldest old*" OR AB "senium" OR AB "very old*" OR AB "septuagenarian*" OR AB "octagenarian*" OR AB "nonagenarian*" OR AB "centarian*" OR AB "centenarian*" OR AB "supercentenarian*" OR AB "older subject*" OR AB "older patient*" OR AB "older age*" OR AB "older men" OR AB "older male*" OR AB "older woman" OR AB "older women" OR AB "older female*" OR AB "older population*" OR AB "older person*")

AND

**Section 2A:**

(TI "Group homes" OR TI "Group living" OR TI "Group dwelling*" OR TI "Collective living" OR TI "Homelike" OR TI "Home like" OR TI "Small scale" OR TI "Smallscale" OR TI "Small unit*" OR TI "Care farm*" OR TI "Green care" OR TI "Green dementia care" OR TI "Social farm*" OR TI "Green hous*" OR TI "dementia friendly" OR TI "homeshar*" OR TI "Dementia villag*" OR TI "Cohous*" OR TI "shared hous*" OR TI "communal liv*" OR AB "Group homes" OR AB "Group living" OR AB "Group dwelling*" OR AB "Collective living" OR AB "Homelike" OR AB "Home like" OR AB "Small scale" OR AB "Smallscale" OR AB "Small unit*" OR AB "Care farm*" OR AB "Green care" OR AB "Green dementia care" OR AB "Social farm*" OR AB "Green hous*" OR AB "dementia friendly" OR AB "homeshar*" OR AB "Dementia villag*" OR AB "Cohous*" OR AB "shared hous*" OR AB "communal liv*")

OR

**Section 2B:**

(MH "Housing for the Elderly" OR MH "Institutionalization+" OR MH "Long Term Care" OR MH "Gerontologic Nursing+" OR MH "Residential Facilities+" OR MH "Nursing Home Design and Construction" OR TI "24-hour care facilit*" OR TI "care home*" OR TI "care institution*" OR TI "extended care facilit*" OR TI "geriatric center*" OR TI "geriatric centre*" OR TI "geriatric facilit*" OR TI "geriatric home*" OR TI "geriatric institution*" OR TI "geriatric unit*" OR TI "group home*" OR TI "home for the aged" OR TI "institutionalized elderly" OR TI "intermediate care facilit*" OR TI "long term care" OR TI "LTCF*" OR TI "nursing center*" OR TI "nursing centre*" OR TI "nursing facilit*" OR TI "nursing home*" OR TI "nursing institution*" OR TI "nursing unit*" OR TI "old age home*" OR TI "residential care" OR TI "residential facilit*" OR TI "skilled nursing facilit*" OR TI "Homes for the elderly" OR TI "Home for the elderly" OR TI "Home for elderly" OR TI "Homes for elderly" OR TI "assisted living facilit*" OR TI "continuing care retirement communit*" OR TI "old folks home*" OR TI "old folks' home*" OR TI "old people's home*" OR TI "rest home*" OR TI "retirement facilit*" OR TI "retirement home*" OR TI "age friendly communit*" OR TI "Livable communit*" OR TI "Intergenerational living" OR TI "Intergenerational housing" OR TI "Senior hous*" OR TI "special care unit*" OR TI "Special care facilit*" OR AB "24-hour care facilit*" OR AB "care home*" OR AB "care institution*" OR AB "extended care facilit*" OR AB "geriatric center*" OR AB "geriatric centre*" OR AB "geriatric facilit*" OR AB "geriatric home*" OR AB "geriatric institution*" OR AB "geriatric unit*" OR AB "group home*" OR AB "home for the aged" OR AB "institutionalized elderly" OR AB "intermediate care facilit*" OR AB "long term care" OR AB "LTCF*" OR AB "nursing center*" OR AB "nursing centre*" OR AB "nursing facilit*" OR AB "nursing home*" OR AB "nursing institution*" OR AB "nursing unit*" OR AB "old age home*" OR AB "residential care" OR AB "residential facilit*" OR AB "skilled nursing facilit*" OR AB "Homes for the elderly" OR AB "Home for the elderly" OR AB "Home for elderly" OR AB "Homes for elderly" OR AB "assisted living facilit*" OR AB "continuing care retirement communit*" OR AB "old folks home*" OR AB "old folks' home*" OR AB "old people's home*" OR AB "rest home*" OR AB "retirement facilit*" OR AB "retirement home*" OR AB "age friendly communit*" OR AB "Livable communit*" OR AB "Intergenerational living" OR AB "Intergenerational housing" OR AB "Senior hous*" OR AB "special care unit*" OR AB "Special care facilit*")

AND

(MH "Diffusion of Innovation+" OR MH "Knowledge Management+" OR MH "Health Care Reform+" OR TI "innovat*" OR TI "Original" OR TI "visionar*" OR TI "chang*" or TI "alternative*" OR TI "modernization*" OR TI "modernisation*" OR TI "cutting edge" OR TI "leading edge" OR TI "inventi*" OR TI "Ingenious" OR TI "reform*")

**Search string EBSCOHOST: PsycInfo**

**Section 1:**

(DE "Aging" OR DE "Cognitive Aging" OR DE "Physiological Aging" OR DE "Dementia" OR DE "Geriatrics" OR DE "Geriatric Psychiatry" OR DE "Gerontology" OR TI "Old adult*" OR TI "Old people" OR TI "Older adult*" OR TI "Older people" OR TI "elder*" OR TI "Dementia" OR TI "Senior*" OR TI "Geriatric*" OR TI "ages 65" OR TI "aged 65" OR TI "65 and over" OR TI "ages 70" OR TI "aged 70" OR TI "70 and over" OR TI "ages 75" OR TI "aged 75" OR TI "75 and over" OR TI "ages 80" OR TI "aged 80" OR TI "80 and over" OR TI "ages 85" OR TI "aged 85" OR TI "85 and over" OR TI "ages 90" OR TI "aged 90" OR TI "90 and over" OR TI "eldest" OR TI "frail*" OR TI "geriatri*" OR TI "old age*" OR TI "oldest old*" OR TI "senium" OR TI "very old*" OR TI "septuagenarian*" OR TI "octagenarian*" OR TI "nonagenarian*" OR TI "centarian*" OR TI "centenarian*" OR TI "supercentenarian*" OR TI "older subject*" OR TI "older patient*" OR TI "older age*" OR TI "older men" OR TI "older male*" OR TI "older woman" OR TI "older women" OR TI "older female*" OR TI "older population*" OR TI "older person*" OR AB "Old adult*" OR AB "Old people" OR AB "Older adult*" OR AB "Older people" OR AB "elder*" OR AB "Dementia" OR AB "Senior*" OR AB "Geriatric*" OR AB "ages 65" OR AB "aged 65" OR AB "65 and over" OR AB "ages 70" OR AB "aged 70" OR AB "70 and over" OR AB "ages 75" OR AB "aged 75" OR AB "75 and over" OR AB "ages 80" OR AB "aged 80" OR AB "80 and over" OR AB "ages 85" OR AB "aged 85" OR AB "85 and over" OR AB "ages 90" OR AB "aged 90" OR AB "90 and over" OR AB "eldest" OR AB "frail*" OR AB "geriatri*" OR AB "old age*" OR AB "oldest old*" OR AB "senium" OR AB "very old*" OR AB "septuagenarian*" OR AB "octagenarian*" OR AB "nonagenarian*" OR AB "centarian*" OR AB "centenarian*" OR AB "supercentenarian*" OR AB "older subject*" OR AB "older patient*" OR AB "older age*" OR AB "older men" OR AB "older male*" OR AB "older woman" OR AB "older women" OR AB "older female*" OR AB "older population*" OR AB "older person*")

AND

**Section 2A:**

(DE "Group Homes" OR TI "Group homes" OR TI "Group living" OR TI "Group dwelling*" OR TI "Collective living" OR TI "Homelike" OR TI "Home like" OR TI "Small scale" OR TI "Smallscale" OR TI "Small unit*" OR TI "Care farm*" OR TI "Green care" OR TI "Green dementia care" OR TI "Social farm*" OR TI "Green hous*" OR TI "dementia friendly" OR TI "homeshar*" OR TI "Dementia villag*" OR TI "Cohous*" OR TI "shared hous*" OR TI "communal liv*" OR AB "Group homes" OR AB "Group living" OR AB "Group dwelling*" OR AB "Collective living" OR AB "Homelike" OR AB "Home like" OR AB "Small scale" OR AB "Smallscale" OR AB "Small unit*" OR AB "Care farm*" OR AB "Green care" OR AB "Green dementia care" OR AB "Social farm*" OR AB "Green hous*" OR AB "dementia friendly" OR AB "homeshar*" OR AB "Dementia villag*" OR AB "Cohous*" OR AB "shared hous*" OR AB "communal liv*")

OR

**Section 2B:**

(DE "Institutionalization" OR DE "Long Term Care" OR DE "Residential Care Institutions" OR DE "Nursing Homes" OR TI "24-hour care facilit*" OR TI "care home*" OR TI "care institution*" OR TI "extended care facilit*" OR TI "geriatric center*" OR TI "geriatric centre*" OR TI "geriatric facilit*" OR TI "geriatric home*" OR TI "geriatric institution*" OR TI "geriatric unit*" OR TI "group home*" OR TI "home for the aged" OR TI "institutionalized elderly" OR TI "intermediate care facilit*" OR TI "long term care" OR TI "LTCF*" OR TI "nursing center*" OR TI "nursing centre*" OR TI "nursing facilit*" OR TI "nursing home*" OR TI "nursing institution*" OR TI "nursing unit*" OR TI "old age home*" OR TI "residential care" OR TI "residential facilit*" OR TI "skilled nursing facilit*" OR TI "Homes for the elderly" OR TI "Home for the elderly" OR TI "Home for elderly" OR TI "Homes for elderly" OR TI "assisted living facilit*" OR TI "continuing care retirement communit*" OR TI "old folks home*" OR TI "old folks' home*" OR TI "old people's home*" OR TI "rest home*" OR TI "retirement facilit*" OR TI "retirement home*" OR TI "age friendly communit*" OR TI "Livable communit*" OR TI "Intergenerational living" OR TI "Intergenerational housing" OR TI "Senior hous*" OR TI "special care unit*" OR TI "Special care facilit*" OR AB "24-hour care facilit*" OR AB "care home*" OR AB "care institution*" OR AB "extended care facilit*" OR AB "geriatric center*" OR AB "geriatric centre*" OR AB "geriatric facilit*" OR AB "geriatric home*" OR AB "geriatric institution*" OR AB "geriatric unit*" OR AB "group home*" OR AB "home for the aged" OR AB "institutionalized elderly" OR AB "intermediate care facilit*" OR AB "long term care" OR AB "LTCF*" OR AB "nursing center*" OR AB "nursing centre*" OR AB "nursing facilit*" OR AB "nursing home*" OR AB "nursing institution*" OR AB "nursing unit*" OR AB "old age home*" OR AB "residential care" OR AB "residential facilit*" OR AB "skilled nursing facilit*" OR AB "Homes for the elderly" OR AB "Home for the elderly" OR AB "Home for elderly" OR AB "Homes for elderly" OR AB "assisted living facilit*" OR AB "continuing care retirement communit*" OR AB "old folks home*" OR AB "old folks' home*" OR AB "old people's home*" OR AB "rest home*" OR AB "retirement facilit*" OR AB "retirement home*" OR AB "age friendly communit*" OR AB "Livable communit*" OR AB "Intergenerational living" OR AB "Intergenerational housing" OR AB "Senior hous*" OR AB "special care unit*" OR AB "Special care facilit*")

AND

(DE "Innovation" OR DE "Knowledge Management" OR DE "Health Care Reform" OR TI "innovat*" OR TI "Original" OR TI "visionar*" OR TI "chang*" or TI "alternative*" OR TI "modernization*" OR TI "modernisation*" OR TI "cutting edge" OR TI "leading edge" OR TI "inventi*" OR TI "Ingenious" OR TI "reform*")

**Search string Web of Science**

**Section 1:**

AB=("Aged" OR "Dementia" OR "Health services for the aged" OR "Geriatrics" OR "Geriatric Psychiatry" OR "Geriatric Nursing" OR "Old adult*" OR "Old people" OR "Older adult*" OR "Older people" OR "elder*" OR "Dementia" OR "Senior*" OR "Geriatric*" OR "ages 65" OR "aged 65" OR "65 and over" OR "ages 70" OR "aged 70" OR "70 and over" OR "ages 75" OR "aged 75" OR "75 and over" OR "ages 80" OR "aged 80" OR "80 and over" OR "ages 85" OR "aged 85" OR "85 and over" OR "ages 90" OR "aged 90" OR "90 and over" OR "elde*" OR "frail*" OR "geriatri*" OR "old age*" OR "oldest old*" OR "senium" OR "very old*" OR "septuagenarian*" OR "octagenarian*" OR "nonagenarian*" OR "centarian*" OR "centenarian*" OR "supercentenarian*" OR "older subject*" OR "older patient*" OR "older age*" OR "older men" OR "older male*" OR "older woman" OR "older women" OR "older female*" OR "older population*" OR "older person*") OR TI=("Aged" OR "Dementia" OR "Health services for the aged" OR "Geriatrics" OR "Geriatric Psychiatry" OR "Geriatric Nursing" OR "Old adult*" OR "Old people" OR "Older adult*" OR "Older people" OR "elder*" OR "Dementia" OR "Senior*" OR "Geriatric*" OR "ages 65" OR "aged 65" OR "65 and over" OR "ages 70" OR "aged 70" OR "70 and over" OR "ages 75" OR "aged 75" OR "75 and over" OR "ages 80" OR "aged 80" OR "80 and over" OR "ages 85" OR "aged 85" OR "85 and over" OR "ages 90" OR "aged 90" OR "90 and over" OR "elde*" OR "frail*" OR "geriatri*" OR "old age*" OR "oldest old*" OR "senium" OR "very old*" OR "septuagenarian*" OR "octagenarian*" OR "nonagenarian*" OR "centarian*" OR "centenarian*" OR "supercentenarian*" OR "older subject*" OR "older patient*" OR "older age*" OR "older men" OR "older male*" OR "older woman" OR "older women" OR "older female*" OR "older population*" OR "older person*")

AND

**Section 2A:**

AB=("Group homes" OR "Group living" OR "Group dwelling*" OR "Collective living" OR "Homelike" OR "Home like" OR "Small scale" OR "Smallscale" OR "Small unit*" OR "Care farm*" OR "Green care" OR "Green dementia care" OR "Social farm*" OR "Green hous*" OR "dementia friendly" OR "homeshar*" OR "Dementia villag*" OR "Cohous*" OR "shared hous*" OR "communal liv*") OR TI=("Group homes" OR "Group living" OR "Group dwelling*" OR "Collective living" OR "Homelike" OR "Home like" OR "Small scale" OR "Smallscale" OR "Small unit*" OR "Care farm*" OR "Green care" OR "Green dementia care" OR "Social farm*" OR "Green hous*" OR "dementia friendly" OR "homeshar*" OR "Dementia villag*" OR "Cohous*" OR "shared hous*" OR "communal liv*")

OR

**Section 2B:**

AB=("Housing for the Elderly" OR "Institutionalization" OR "Long-Term Care" OR "Geriatric Nursing" OR "Residential Facilities" OR "Environment Design" OR "Nursing homes" OR "24-hour care facilit*" OR "care home*" OR "care institution*" OR "extended care facilit*" OR "geriatric center*" OR "geriatric centre*" OR "geriatric facilit*" OR "geriatric home*" OR "geriatric institution*" OR "geriatric unit*" OR "group home*" OR "home for the aged" OR "institutionalized elderly" OR "intermediate care facilit*" OR "long term care" OR "LTCF*" OR "nursing center*" OR "nursing centre*" OR "nursing facilit*" OR "nursing home*" OR "nursing institution*" OR "nursing unit*" OR "old age home*" OR "residential care" OR "residential facilit*" OR "skilled nursing facilit*" OR "Homes for the elderly" OR "Home for the elderly" OR "Home for elderly" OR "Homes for elderly" OR "assisted living facilit*" OR "continuing care retirement communit*" OR "old folks home*" OR "old folks' home*" OR "old people's home*" OR "rest home*" OR "retirement facilit*" OR "retirement home*" OR "age friendly communit*" OR "Livable communit*" OR "Intergenerational living" OR "Intergenerational housing" OR "Senior hous*" OR "special care unit*" OR "Special care facilit*") OR TI=("Housing for the Elderly" OR "Institutionalization" OR "Long-Term Care" OR "Geriatric Nursing" OR "Residential Facilities" OR "Environment Design" OR "Nursing homes" OR "24-hour care facilit*" OR "care home*" OR "care institution*" OR "extended care facilit*" OR "geriatric center*" OR "geriatric centre*" OR "geriatric facilit*" OR "geriatric home*" OR "geriatric institution*" OR "geriatric unit*" OR "group home*" OR "home for the aged" OR "institutionalized elderly" OR "intermediate care facilit*" OR "long term care" OR "LTCF*" OR "nursing center*" OR "nursing centre*" OR "nursing facilit*" OR "nursing home*" OR "nursing institution*" OR "nursing unit*" OR "old age home*" OR "residential care" OR "residential facilit*" OR "skilled nursing facilit*" OR "Homes for the elderly" OR "Home for the elderly" OR "Home for elderly" OR "Homes for elderly" OR "assisted living facilit*" OR "continuing care retirement communit*" OR "old folks home*" OR "old folks' home*" OR "old people's home*" OR "rest home*" OR "retirement facilit*" OR "retirement home*" OR "age friendly communit*" OR "Livable communit*" OR "Intergenerational living" OR "Intergenerational housing" OR "Senior hous*" OR "special care unit*" OR "Special care facilit*")

AND

TI=("Organizational innovation" OR "Diffusion of Innovation" OR "Inventions" OR "Knowledge Management" OR "Health Care Reform" OR "innovat*" OR "Original" OR "visionar*" OR "chang*" or "alternative*" OR "modernization*" OR "modernisation*" OR "cutting edge" OR "leading edge" OR "inventi*" OR "Ingenious" OR "reform*")
